# Hypereosinophilic syndrome with Löffler endocarditis and multiple cerebral infarctions in a patient with a CLL-phenotype clonal B-cell population: a case report

**DOI:** 10.3389/fcvm.2026.1924869

**Published:** 2026-08-19

**Authors:** Haopeng Wang, Fu Zhu, Hailing Hu

**Affiliations:** Department of Cardiovascular Disease, Weifang Traditional Chinese Hospital, Weifang, China

**Keywords:** cardiac magnetic resonance, cerebral infarction, chronic lymphocytic leukemia, hypereosinophilic syndrome, Löffler's endocarditis

## Abstract

Hypereosinophilic syndrome (HES) is defined by marked eosinophilia accompanied by eosinophil-mediated organ injury. Cardiac involvement can progress from endomyocardial inflammation to mural thrombosis and fibrosis, with systemic embolization. A 58-year-old man presented with recurrent chest distress and back pain. Laboratory testing showed an absolute eosinophil count of 2.01 × 10⁹/L, cardiac troponin I of 0.25 ng/mL, N-terminal pro-B-type natriuretic peptide of 3,272 pg/mL, and D-dimer of 1.12 mg/L. Coronary angiography excluded fixed obstructive coronary disease and demonstrated dynamic systolic compression of the mid-left anterior descending artery due to myocardial bridging. Brain magnetic resonance imaging, performed because of memory decline and concern for clinically silent embolization, identified multifocal acute cerebral infarctions. Cardiac magnetic resonance imaging demonstrated left ventricular endocardial thickening, subendocardial late gadolinium enhancement, and a non-enhancing mural thrombus, consistent with Löffler endocarditis. Bone marrow eosinophils accounted for 56.4% of nucleated cells. Flow cytometry identified a CLL-phenotype clonal mature B-cell population; however, the peripheral blood CD19-positive B-cell count was only 0.334 × 10⁹/L and no lymphadenopathy or hepatosplenomegaly was found, making overt chronic lymphocytic leukemia unsupported by current criteria. A limited myeloid fusion panel was negative. The patient received dexamethasone 20 mg/day and anticoagulation with enoxaparin and warfarin. The eosinophil count normalized within four days, but six-month cardiac magnetic resonance follow-up showed persistent endocardial disease and a slightly enlarged mural thrombus. This case highlights the need for integrated hematologic, immunologic, and cardiac evaluation in HES, cautious interpretation of coincident lymphoid clones, and long-term imaging and anticoagulation surveillance even after rapid hematologic improvement.

## 1 Introduction

Hypereosinophilia is generally defined as a peripheral blood absolute eosinophil count of at least 1.5 × 10⁹/L. When eosinophilia is accompanied by attributable tissue or organ injury, the condition is termed hypereosinophilic syndrome (HES) (1–3). HES encompasses secondary or reactive disorders, primary or clonal eosinophilic neoplasms, lymphocytic-variant disease, and cases in which no cause is established. An integrated evaluation of exposure history, morphology, immunophenotype, cytogenetics, and molecular testing is therefore essential (1, 4).

Cardiac involvement is among the most serious manifestations of HES. Eosinophil-mediated endomyocardial injury may evolve through inflammatory, thrombotic, and fibrotic stages, producing Löffler endocarditis, restrictive physiology, intracardiac thrombi, and systemic embolization (3, 5). Cerebral infarction may result from cardiac embolism, eosinophil-related endothelial injury, or low-flow mechanisms (5).

Lymphocytic-variant HES is most often associated with aberrant T-cell populations that produce eosinophilopoietic cytokines. Associations with mature B-cell clones are much less clearly established. Moreover, a CLL-like immunophenotype does not itself establish chronic lymphocytic leukemia (CLL); current diagnostic criteria require an adequate peripheral blood clonal B-cell count or other defining tissue or marrow features (6, 7). We report a patient with HES, Löffler endocarditis, left ventricular mural thrombosis, and multifocal cerebral infarctions in whom a low-count CLL-phenotype clonal B-cell population was identified. The case emphasizes both the severity of eosinophil-mediated cardiac disease and the need to avoid over-attributing HES to an incidental lymphoid clone.

## 2 Case presentation

### 2.1 Initial presentation

A 58-year-old man with hyperlipidemia and a long smoking history was admitted on 3 October 2025 because of recurrent episodes of chest distress and back pain for approximately two weeks. Each episode lasted about 10 min. He denied fever, rash, asthma, allergic rhinitis, recent initiation of a regular medication, and known drug allergy. No clear travel, raw-food, or parasitic exposure history was documented. He reported a decline in memory over the preceding six months but had no focal neurologic deficit on admission. Physical examination showed a temperature of 36.2 °C, blood pressure of 118/81 mmHg, heart rate of 76 beats/min, and no palpable lymphadenopathy, edema, hepatomegaly, or splenomegaly.

The initial white blood cell count was 11.58 × 10⁹/L, with an absolute eosinophil count of 2.01 × 10⁹/L (17.4%) and an absolute lymphocyte count of 2.45 × 10⁹/L. Hemoglobin was 116 g/L with microcytosis (mean corpuscular volume, 70.2 fL) and increased red-cell distribution width; platelets were 130 × 10⁹/L. Cardiac troponin I was 0.25 ng/mL, N-terminal pro-B-type natriuretic peptide was 3,272 pg/mL, and D-dimer was 1.12 mg/L. Fibrinogen was 1.51 g/L. Renal and hepatic function were preserved. Electrocardiography showed sinus rhythm with left-axis deviation and diffuse ST-T abnormalities.

### 2.2 Cardiovascular and neurologic imaging

**Figure 1:**
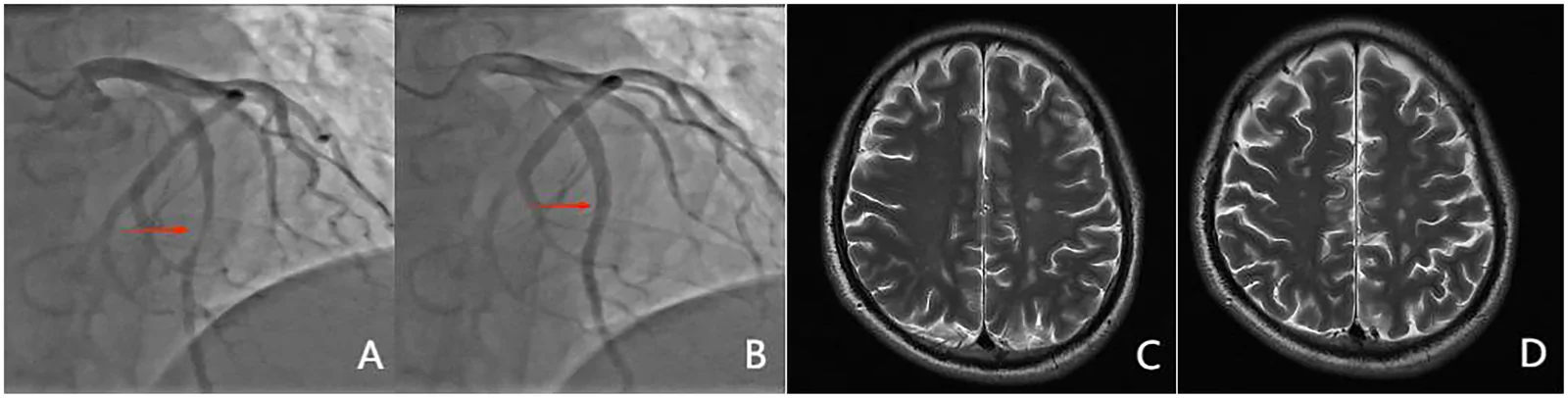
Coronary angiography and brain magnetic resonance imaging. **(A)** A systolic coronary angiographic frame shows approximately 80% dynamic compression of the mid-left anterior descending artery due to myocardial bridging (red arrow), without a fixed obstructive lesion. **(B)** Diastolic coronary angiography showed no compression of the mid-portion of the left anterior descending artery (red arrow). **(C,D)** Axial T2-weighted brain MRI images show multifocal hyperintense infarct lesions in the bilateral frontoparietal regions.

Emergency coronary angiography demonstrated no fixed obstructive coronary lesion. A systolic angiographic frame showed approximately 80% dynamic compression of the mid-left anterior descending artery, compatible with myocardial bridging (Figures 1A,B). The dynamic systolic narrowing, rather than a persistent lesion throughout the cardiac cycle, argued against fixed atherosclerotic stenosis as the explanation for the overall presentation.

Because of persistent symptoms, markedly increased natriuretic peptide levels, memory decline, hypereosinophilia, and concern for intracardiac thrombosis, further imaging was performed. Brain magnetic resonance imaging, including diffusion-weighted imaging as part of the complete examination, showed multiple acute infarctions involving the bilateral frontoparietal regions (Figures 1C,D). Pulmonary vascular imaging showed no pulmonary embolism.

**Figure 2:**
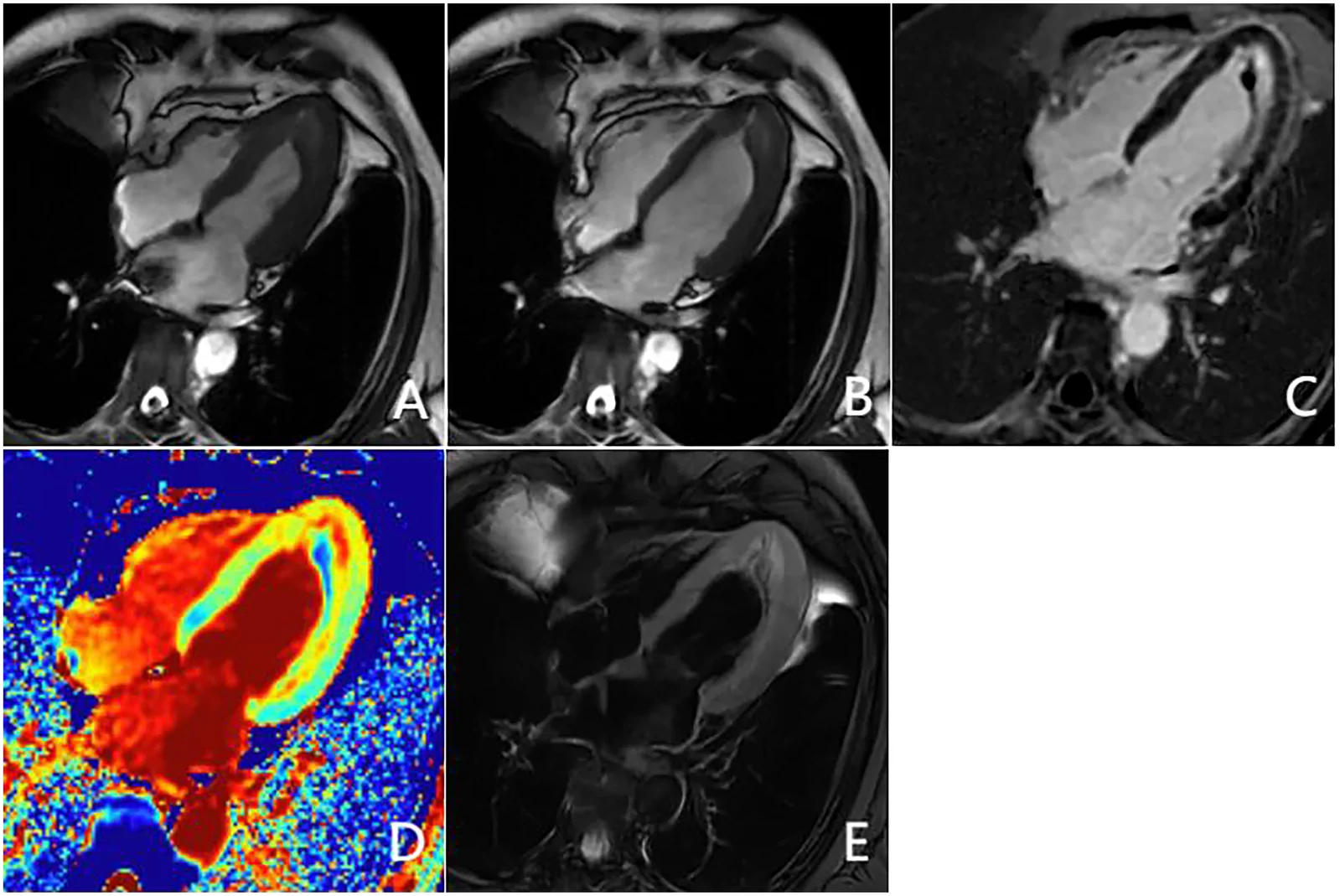
Cardiac magnetic resonance findings of Löffler endocarditis with mural thrombus. **(A,B)** Four-chamber cine images in systole and diastole show abnormal left ventricular endocardial thickening and an apical intracavitary filling defect. **(C)** Phase-sensitive inversion recovery late gadolinium enhancement imaging shows subendocardial enhancement with an adjacent non-enhancing intracavitary layer representing mural thrombus. **(D)** T1 mapping demonstrates abnormal signal characteristics in the involved endomyocardium. **(E)** Fat-suppressed T2-weighted imaging shows increased signal in the involved endomyocardial region, consistent with active edema or inflammation.

Cardiac magnetic resonance imaging demonstrated abnormal left ventricular endocardial thickening and an apical intracavitary filling defect on cine images. Phase-sensitive inversion recovery imaging showed subendocardial late gadolinium enhancement adjacent to a non-enhancing intracavitary layer, compatible with mural thrombus. T1 mapping and fat-suppressed T2-weighted imaging showed abnormal signal in the involved endomyocardium, supporting active eosinophilic endomyocardial injury with thrombosis (Figures 2A–E). The integrated findings were considered diagnostic of Löffler endocarditis with left ventricular mural thrombus.

### 2.3 Hematologic and etiologic evaluation

**Figure 3:**
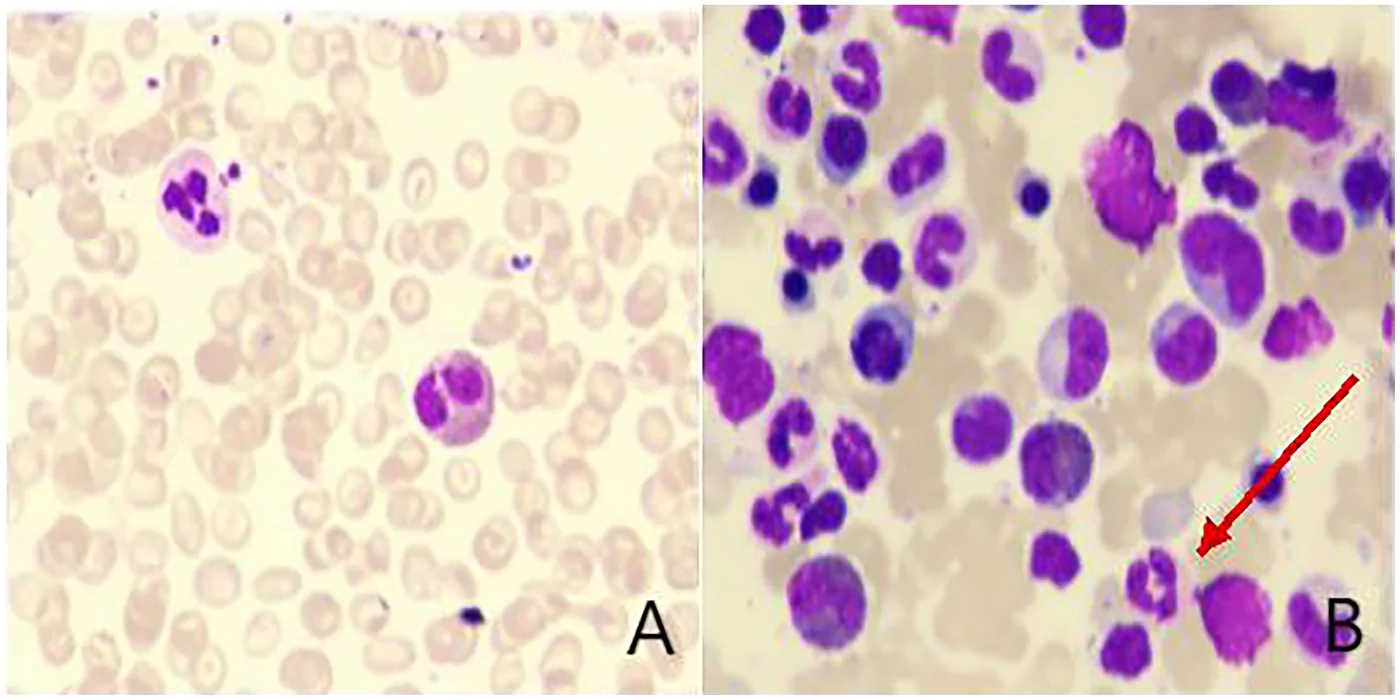
Peripheral blood and bone marrow morphology. **(A)** Peripheral blood smear (May-Grünwald-Giemsa stain, ×100 objective) shows increased eosinophils, accounting for approximately 25% of leukocytes. **(B)** Bone marrow aspirate smear (May-Grünwald-Giemsa stain, ×100 objective) shows marked eosinophilic hyperplasia; eosinophils comprised 56.4% of nucleated marrow cells. The red arrow indicates a representative eosinophil.

Serial blood counts confirmed persistent hypereosinophilia, with the absolute eosinophil count reaching 2.16 × 10⁹/L. Peripheral blood smear examination showed eosinophils accounting for approximately 25% of leukocytes, without circulating blasts. Bone marrow aspiration showed active trilineage hematopoiesis and striking eosinophilic hyperplasia; eosinophils comprised 56.4% of nucleated marrow cells. The report did not describe blast excess, overt multilineage dysplasia, or abnormal megakaryocytic morphology (Figures 3A,B).

Bone marrow flow cytometry identified an aberrant clonal mature B-cell population accounting for 14.89% of nucleated cells. The cells expressed CD19, CD5, CD23, CD200, and dim CD43, showed weak kappa light-chain restriction, and were negative for CD10, FMC7, and CD79b, yielding a CLL immunophenotypic score of 5. However, the peripheral blood absolute lymphocyte counts were 2.45 and 2.21 × 10⁹/L on serial testing, and the absolute CD19-positive B-cell count was 334/µL (0.334 × 10⁹/L). No clinically significant lymphadenopathy or hepatosplenomegaly was identified, and the microcytic anemia was not attributable to a typical clonal B-cell marrow infiltrate. Accordingly, the findings did not satisfy current diagnostic criteria for CLL or small lymphocytic lymphoma and were considered most consistent with a low-count CLL-phenotype clonal B-cell population/monoclonal B-cell lymphocytosis (6, 7). Rai and Binet staging were therefore not assigned. CLL-associated FISH, TP53 mutation, and IGHV mutation testing were not performed.

A screening panel for recurrent fusion genes associated with clonal myeloid eosinophilic disorders was negative, including CBFB::MYH11, FIP1L1::PDGFRA, ETV6::PDGFRB, ETV6::ABL1, PCM1::JAK2, BCR::FGFR1, and ZNF198::FGFR1. The negative panel reduced the likelihood of a defined myeloid/lymphoid neoplasm with a tested tyrosine kinase fusion but did not prove that the eosinophilia was non-clonal.

Testing for MPO-ANCA, PR3-ANCA, anti-glomerular basement membrane antibodies, rheumatoid factor, and antiphospholipid antibodies was negative. Serum IgG4 and total IgG, IgA, and IgM were within reference ranges. In contrast, autoimmune screening showed low-level ANA positivity (42.1 AU/mL), increased anti-double-stranded DNA antibody (68.3 IU/mL), markedly increased anti-nucleosome antibody (>625 AU/mL on repeat testing), and mildly decreased C3 (0.74 g/L). The patient lacked sufficient clinical manifestations to establish a defined connective tissue disease. Stool ova and parasite testing, Strongyloides serology, expanded aberrant T-cell immunophenotyping, T-cell receptor gene rearrangement, conventional cytogenetics, comprehensive myeloid next-generation sequencing, and marrow fibrosis assessment were not available. The final working diagnosis was therefore HES of uncertain etiology, complicated by Löffler endocarditis, left ventricular mural thrombus, and multifocal cerebral infarctions, with a concomitant low-count CLL-phenotype clonal B-cell population.

### 2.4 Treatment and follow-up

Following multidisciplinary consultation, treatment focused on the organ-threatening eosinophilic and thromboembolic complications. Dexamethasone 20 mg/day was initiated. Because of the left ventricular mural thrombus and multifocal cerebral infarctions, anticoagulation with enoxaparin and warfarin was administered, with serial monitoring of prothrombin time and the international normalized ratio. The exact enoxaparin dose, warfarin initiation schedule, bridging duration, target international normalized ratio, and post-discharge time in therapeutic range could not be fully reconstructed from the available records.

The eosinophil count declined rapidly and returned to the reference range within four days. Cardiac troponin I decreased to 0.08 ng/mL, whereas N-terminal pro-B-type natriuretic peptide remained markedly increased at approximately 3002 pg/mL during hospitalization. No B-cell clone-directed therapy was initiated. The patient was discharged after 24 days. The detailed outpatient corticosteroid taper and continuous post-discharge eosinophil counts were unavailable.

At approximately six months, cardiac magnetic resonance imaging on 21 April 2026 showed persistent endocardial thickening involving the mid-to-distal anterior, lateral, and inferior left ventricular walls and the apex, with corresponding abnormal T1/T2 signal, perfusion defects, and endocardial enhancement. A non-enhancing mural thrombus remained present and was reported to be slightly larger than on the initial examination. Global left ventricular systolic function was preserved (left ventricular ejection fraction, 59.6%; end-diastolic volume, 149.8 mL; end-systolic volume, 60.5 mL; cardiac output, 5.71 L/min), but diastolic function was impaired. Thus, the early hematologic response was not accompanied by clear structural cardiac remission. The overall clinical course is summarized in Table 1.

**Table 1:** Clinical timeline.

Date	Clinical event
3 October 2025	Admission for recurrent chest distress and back pain; AEC 2.01 × 10⁹/L, troponin I 0.25 ng/mL, NT-proBNP 3,272 pg/mL, D-dimer 1.12 mg/L.
3 October 2025	Coronary angiography: no fixed obstructive coronary disease; approximately 80% systolic compression of the mid-LAD due to myocardial bridging.
8–9 October 2025	Cardiac MRI: Löffler endocarditis with mural thrombus. Brain MRI: multifocal acute cerebral infarctions.
9–10 October 2025	Peripheral blood eosinophils approximately 25%; bone marrow eosinophils 56.4%; CLL-phenotype clonal B-cell population detected. Myeloid fusion panel negative.
October 2025	Dexamethasone 20 mg/day and anticoagulation with enoxaparin/warfarin initiated; eosinophils normalized within four days.
27 October 2025	Discharged after 24 days; no clone-directed B-cell therapy initiated.
21 April 2026	Follow-up cardiac MRI: persistent endocardial disease, preserved LVEF (59.6%), impaired diastolic function, and slightly enlarged mural thrombus.

## 3 Discussion

### 3.1 Cardiac and neurologic manifestations of HES

This case illustrates the thrombotic stage of eosinophilic endomyocardial disease. Activated eosinophils release cytotoxic granule proteins and inflammatory mediators that injure the endocardium and myocardium, creating a thrombogenic surface and subsequently promoting fibrosis (1, 3). Cardiac magnetic resonance is particularly useful because it can simultaneously demonstrate endocardial thickening, edema, subendocardial late gadolinium enhancement, perfusion abnormalities, and non-enhancing thrombus. When enhanced endocardium and blood pool are separated by a non-enhancing thrombotic layer, a characteristic layered or “sandwich” appearance may be seen (8).

Multifocal cerebral infarction is a recognized but uncommon neurologic complication of HES. The distribution may reflect embolization from an intracardiac thrombus, eosinophil-mediated endothelial injury, microvascular thrombosis, or watershed hypoperfusion (5, 9). In the present patient, the mural thrombus provided a plausible embolic source, while the multifocal pattern and underlying eosinophilic vasculopathy may also have contributed. The absence of a focal neurologic deficit does not exclude clinically relevant cerebral injury; memory decline and the high embolic-risk cardiac phenotype justified brain magnetic resonance imaging.

### 3.2 Interpreting the CLL-phenotype B-cell clone

The original diagnostic interpretation of CLL required revision. Current iwCLL criteria require at least 5 × 10⁹/L clonal B lymphocytes in peripheral blood sustained for at least three months, unless a typical marrow infiltrate causes otherwise unexplained cytopenia; small lymphocytic lymphoma requires tissue involvement (6). WHO classification recognizes low-count monoclonal B-cell lymphocytosis when a CLL-phenotype clonal B-cell count is below 0.5 × 10⁹/L without lymphadenopathy, organomegaly, or other defining disease features (7). In this patient, the total circulating CD19-positive B-cell count was only 0.334 × 10⁹/L, and no defining tissue disease was identified. The most defensible classification is therefore a low-count CLL-phenotype clonal B-cell population rather than overt CLL (10).

The relationship between this clone and the eosinophilia remains uncertain. Mature B-cell neoplasms may alter the cytokine milieu, but direct production of interleukin-5 by the detected clone was not measured. The rapid steroid response is compatible with reactive or idiopathic HES but is not specific for a B-cell-mediated mechanism. Accordingly, the clone may have contributed to the eosinophilia, may have served as a marker of immune dysregulation, or may have been incidental. The manuscript therefore avoids describing the B-cell clone as the proven driver of HES.

### 3.3 Differential diagnosis

Secondary causes of eosinophilia require systematic exclusion. The absence of rash, asthma, a clear new-drug exposure, or a typical parasitic exposure reduced the likelihood of common reactive causes, but dedicated stool and Strongyloides testing were not performed. The abnormal ANA, anti-double-stranded DNA, anti-nucleosome antibody, and C3 results raise the possibility of immune dysregulation; however, no defined connective tissue disease could be established clinically. Negative ANCA results and the absence of asthma, vasculitic skin disease, mononeuritis, or renal injury made eosinophilic granulomatosis with polyangiitis less likely but did not absolutely exclude it.

Lymphocytic-variant HES is characterized by aberrant or clonal T cells that produce eosinophilopoietic cytokines. Routine CD3, CD4, and CD8 subset counts are insufficient to exclude this diagnosis. Expanded flow cytometry for aberrant phenotypes, such as CD3-negative/CD4-positive populations or loss of CD7 or CD26, together with T-cell receptor clonality and, when available, TARC/CCL17 or intracellular cytokine assessment, provides greater diagnostic sensitivity (4, 11). Because these studies were not performed, lymphocytic-variant HES remained incompletely excluded.

Chronic eosinophilic leukemia and myeloid/lymphoid neoplasms with eosinophilia also require consideration. The marked marrow eosinophilia was notable, but no blast excess or overt multilineage dysplasia was reported, and the tested recurrent fusion genes were negative. Nevertheless, a limited negative fusion panel does not establish a reactive origin. Contemporary classification relies on integrated marrow morphology, cytogenetics, fluorescence *in situ* hybridization, and broad molecular testing (1, 12). Conventional karyotyping, comprehensive myeloid next-generation sequencing, and fibrosis assessment would have strengthened exclusion of a clonal myeloid process.

### 3.4 Treatment, anticoagulation, and follow-up

Organ-threatening HES warrants prompt eosinophil-lowering therapy, and systemic corticosteroids remain a standard initial approach when an immediately targetable molecular driver is not known (2, 4, 13). In this patient, dexamethasone produced a rapid hematologic response. However, normalization of circulating eosinophils did not translate into resolution of endocardial injury or mural thrombus at six months. The persistent cardiac magnetic resonance abnormalities suggest established thrombotic and fibrotic disease and underscore the importance of serial imaging, cardiac biomarkers, and clinical assessment beyond the initial blood-count response.

Anticoagulation was justified by the combination of left ventricular mural thrombus and multifocal cerebral infarctions. Enoxaparin and warfarin were used with in-hospital PT/INR monitoring. The follow-up finding of a slightly enlarged thrombus raises important questions regarding treatment duration, adherence, and time in therapeutic range, but the available records were insufficient to determine these factors. In comparable cases with molecularly defined chronic eosinophilic leukemia, effective treatment of the driver lesion together with anticoagulation has produced thrombus resolution, whereas fulminant disease may remain fatal despite corticosteroids, cytoreduction, and heparin (9, 14, 15).

### 3.5 Literature context

A focused literature review identified several reports of Löffler endocarditis associated with molecularly defined chronic eosinophilic leukemia, including FIP1L1::PDGFRA-positive disease and a rare JAK2 exon 13 insertion/deletion, but no directly comparable report linking untreated low-count CLL-phenotype monoclonal B-cell lymphocytosis to Löffler endocarditis with multifocal cerebral infarctions was identified (9, 14, 15). Previously published CLL-associated eosinophilia has often been treatment related rather than a clearly demonstrated paraneoplastic HES. The selected cases summarized in Table 2 highlight the importance of defining the hematologic driver because targeted therapy can materially alter cardiac and thrombotic outcomes (9, 14–16).

**Table 2:** Selected hematologic-neoplasm-associated Löffler endocarditis cases.

Study	Patient/hematologic context	Cardiac and neurologic findings	Treatment	Outcome
Kim et al. (14)	28-year-old man; FIP1L1::PDGFRA-positive chronic eosinophilic leukemia	Biventricular endocardial thickening and thrombi; cerebral infarction with neurologic deficits	Imatinib 100 mg/day plus enoxaparin	Hematologic and cytogenetic remission; thrombi and endocardial abnormalities resolved within 3 months; no recurrence over >1 year
Eisenach et al. (9)	50-year-old man; JAK2 exon13InDel-positive chronic eosinophilic leukemia	Bilateral cerebral infarctions; extensive endomyocardial fibrosis; left atrial and mitral-valve thrombi; pulmonary involvement	Prednisone, cytarabine, and intravenous unfractionated heparin	Rapid clinical deterioration with ventricular fibrillation; death 6 days after admission
Faraj et al. (15)	31-year-old patient; FIP1L1::PDGFRA-positive chronic eosinophilic leukemia	Löffler endocarditis with intracardiac thrombi and embolic cerebral infarctions	Imatinib-based therapy/chemotherapy plus anticoagulation	Marked hematologic response and complete thrombus resolution on follow-up cardiac MRI; imatinib dose reduced because of cytopenia
Present case	58-year-old man; HES of uncertain etiology with low-count CLL-phenotype clonal B-cell population	Left ventricular endocardial disease and mural thrombus; multifocal cerebral infarctions	Dexamethasone 20 mg/day; enoxaparin and warfarin; no B-cell clone-directed therapy	Eosinophils normalized within 4 days; persistent cardiac disease and slightly enlarged mural thrombus at approximately 6 months

### 3.6 Limitations

This report has several limitations. First, systematic parasitic testing, expanded aberrant T-cell phenotyping, T-cell receptor clonality, conventional cytogenetics, broad myeloid sequencing, and marrow fibrosis assessment were not available; the cause of HES therefore remains uncertain. Second, the absolute clonal B-cell count was not directly quantified using a dedicated peripheral blood CLL panel, although the total CD19-positive B-cell count was well below the CLL threshold. Third, CLL-associated FISH, TP53, and IGHV testing were not performed because diagnostic criteria for overt CLL were not met. Finally, exact anticoagulant dosing, post-discharge INR control, corticosteroid tapering, and serial outpatient eosinophil counts could not be fully retrieved.

## 4 Conclusion

HES can cause severe eosinophilic endomyocardial disease, mural thrombosis, and clinically subtle but extensive cerebral embolic injury. Cardiac magnetic resonance imaging is valuable for diagnosis and longitudinal assessment, while brain imaging should be considered when intracardiac thrombus or neurologic symptoms raise concern for occult embolization. A concomitant CLL-phenotype B-cell clone should be classified according to absolute clonal B-cell burden and tissue involvement and should not automatically be assumed to cause HES. Rapid normalization of eosinophils does not ensure resolution of established cardiac disease; sustained etiologic evaluation, therapeutic anticoagulation surveillance, and repeated cardiac imaging remain essential.

## Data Availability

The original contributions presented in the study are included in the article/Supplementary Material, further inquiries can be directed to the corresponding author.
